# Plasma levels of an atropine/scopolamine mixture following ingestion of low doses as food contaminant

**DOI:** 10.1186/2050-6511-13-S1-A34

**Published:** 2012-09-17

**Authors:** Lucija Perharič, Gordana Koželj, Lovro Stanovnik

**Affiliations:** 1 Department of Infectious Diseases and Environmental Risks, National Institute of Public Health, 1000 Ljubljana, Slovenia; 2 Institute of Forensic Medicine, Faculty of Medicine, University of Ljubljana, Slovenia; 3 Department of Pharmacology and Experimental Toxicology, Faculty of Medicine, University of Ljubljana, 1000 Ljubljana, Slovenia

## Background

Mass poisoning with *Datura* alkaloids, present in contaminated buckwheat flour occurred in 2003 in Slovenia. A preliminary risk assessment for atropine and scopolamine in the flour was carried out. To obtain more accurate information, we performed a randomised, double-blind, placebo-controlled, crossover study in 20 healthy adult volunteers exposed to low doses of an atropine/scopolamine mixture as food contaminant.

## Methods

The volunteers ingested a traditional Slovenian dish, "žganci", made of boiled buckwheat flour to which 5 doses of an atropine/scopolamine mixture in 2:1 ratio were added. Besides the control meal, the volunteers ingested the following doses of atropine/scopolamine mixture: 0.12 of atropine/0.10 of scopolamine (0.32 expressed as atropine) µg/kg body mass (bm), 0.37/0.29 (0.95) µg/kg bm, 1.22/0.95 (3.12), 3.58/2.81 (9.20) µg/kg bm and 12.10/9.50 (31.10) µg/kg bm. Changes in body temperature, heart rate, saliva and sweat secretion, pupil size and near-point vision as well as subjective symptoms were checked at regular intervals for up to 4 hours after the ingestion. To determine the low levels of each alkaloid in the plasma, samples of venous blood were taken before the ingestion and 30, 60, 120 and 240 minutes afterwards, and analysed by a validated liquid chromatography/tandem mass spectrometry. The study was approved by the national Medical Ethics Committee.

## Results and conclusions

The maximum effects on salivary and sweat secretion were observed at 90 minutes after ingestion and persisted up to 240 minutes. The maximum effect on heart rate was noted at 120 minutes and lasted until 165 minutes, whereas the maximum effect on pupil size and near-point vision was observed at 240 minutes after the ingestion. The maximum concentrations of alkaloids in the plasma were reached 120 minutes after the ingestion, whereas scopolamine had another smaller peak at 30 minutes. The maximum concentrations of atropine and scopolamine after the highest dose were 2.52 ± 0.19 μg/L and 0.49 ± 0.06 μg/L, respectively. Following the ingestion of the low-dose atropine/scopolamine mixture, the area under the curve (AUC_0–4h_) of atropine was 2 to 5 times higher than the AUC_0–4h_ of scopolamine which may have been due to the differences in metabolism. The changes in the heart rate were to some degree associated with the maximum plasma concentrations, whereas the time course of the other endpoints was not.

